# Responsible use of new antibiotics for critically ill patients suffering from infections due to carbapenem-resistant organisms—a narrative review for an enablement strategy

**DOI:** 10.1093/jacamr/dlag226

**Published:** 2026-10-31

**Authors:** Christian Eckmann, Murat Akova, Massimo Antonelli, Matteo Bassetti, Rafael Cantón, Matthew Dryden, Marco Falcone, Souha S Kanj, Sasisopin Kiertiburanakul, Mark Gilchrist, Philippe Montravers, David L Paterson, Eric Senneville, Alex Soriano, Jean-François Timsit, Maria Virginia Villegas, Pierluigi Viale

**Affiliations:** AMS Team and Department of General, Visceral and Thoracic Surgery, Klinikum Hannoversch-Muenden, Goettingen University, Hannoversch-Muenden D-34346, Germany; Department of Infectious Diseases, Hacettepe University School of Medicine, Ankara, Turkey; Department of Emergency, Intensive Care Medicine and Anesthesia, Fondazione Policlinico Universitario A. Gemelli IRCCS, Rome, Italy; Department of Anesthesiology and Intensive Care Medicine, Catholic University of the Sacred Heart, Rome, Italy; Department of Health Sciences (DISSAL), University of Genoa, Genoa, Italy; UO Clinica Malattie Infettive, IRCCS Ospedale Policlinico San Martino, Genoa, Italy; Servicio de Microbiología, Hospital Universitario Ramón y Cajal, Instituto Ramón y Cajal de Investigación Sanitaria (IRYCIS), Madrid, Spain; CIBER de Enfermedades Infecciosas (CIBERINFEC), Instituto de Salud Carlos III, Madrid, Spain; Global Operations, UK Health Security Agency, Porton Down, UK; Department of Clinical and Experimental Medicine, Azienda Ospedaliero Universitaria Pisana, University of Pisa, Pisa, Italy; Division of Infectious Diseases, Infection Control and Prevention Program, Center for Infectious Diseases Research (CIDR), American University of Beirut, Beirut, Lebanon; Faculty of Medicine Ramathibodi Hospital, Mahidol University, Bangkok, Thailand; Department of Infection/Pharmacy, Imperial Healthcare NHS Trust, London, UK; Department of Infectious Disease, Faculty of Medicine, Imperial College London, London, UK; Department of Anaesthesiology and Surgical Intensive Care, Bichat-Claude Bernard, Paris, France; Saw Swee Hock School of Public Health, National University of Singapore and National University Health System, Singapore, Singapore; Discipline of Infectious Diseases, The University of Lille, Gustave Dron Hospital, Tourcoing, France; Infectious Disease Department, Hospital Clínic de Barcelona, University of Barcelona, Barcelona, Spain; IDIBAPS, Barcelona, Spain; CIBERINFEC, Ciber in Infectious Diseases, ISCIII, Madrid, Spain; UMR 1137, Université Paris-Cité, INSERM, IAME, Paris 75018, France; Medical and Infectious Diseases Intensive Care Unit, AP-HP, Bichat-Claude Bernard University Hospital, Paris, France; Grupo de Investigación en Resistencia Antimicrobiana y Epidemiología Hospitalaria (RAEH), Universidad El Bosque, Bogotá, Colombia; Infectious Diseases Unit, Scientific Institute for Research, Hospitalization and Healthcare, Azienda Ospedaliero-Universitaria di Bologna, Bologna, Italy

## Abstract

Infections caused by multidrug-resistant Gram-negative (MDR-GN) bacteria, particularly carbapenem-resistant organisms (CROs), constitute a significant burden in critically ill patients (CIPs). A multidisciplinary, collaborative approach is essential for responsible antibiotic use in CIPs, balancing the necessity for effective treatment with mitigation of antimicrobial resistance (AMR). While the efficacy of existing antibiotics against CROs is increasingly limited due to resistance development and suboptimal pharmacokinetic/pharmacodynamic properties, several barriers impede the widespread adoption of new agents, including selective susceptibility testing, limited availability, high costs, and concerns regarding the fostering of resistance. Antimicrobial stewardship (AMS) strategies aim to optimize antibiotic use; however, there is an ongoing debate between restrictive and enabling approaches for new antibiotics in CIPs with CRO infections. While restrictive strategies focus on limiting antibiotic use to mitigate resistance, they may delay necessary treatment and negatively impact professional culture. Enablement strategies promote informed decision-making and accessibility when needed but require substantial workforce and expertise. In this narrative review and expert opinion, we propose an algorithm supporting a coordinated AMS approach in CIPs, emphasizing the importance of early diagnosis and appropriate therapy. We advocate for an enablement strategy for new antibiotics in CIPs with MDR-GN infections, aiming to reduce mortality and toxicity associated with older agents. This strategy should be integrated into local AMS programmes under the guidance of experienced clinicians.

## Introduction

Antimicrobial resistance (AMR) poses a substantial challenge to public health, economy and healthcare systems worldwide. Clinically, AMR leads to an increased prevalence of infections that are difficult to treat, contributing to higher mortality rates.^1^ This situation is further compounded by reduced cure rates, as standard treatments become less effective, necessitating more complex and prolonged medical interventions.^1^ The recurrence of infections becomes more common, adding to the clinical complexity and the overall health burden.^1^ Economically, AMR escalates costs across the healthcare system. Hospitals face longer lengths of stay (LOS) for patients as they struggle with difficult-to-treat infections, particularly in ICUs.^1^ These prolonged stays not only tie up resources but also lead to increased isolation costs, as measures need to be taken to prevent the spread of resistant strains.^1^ Additionally, there is a financial impact from the need for staff with higher training levels, more specialized care, and the use of more expensive, often last-resort, antimicrobials.^1^ The routine costs of hygiene measures also inflate as more rigorous protocols are required to manage and contain AMR.^1^ The intersection of clinical and economic factors creates a vicious circle where the clinical implications of AMR drive up healthcare costs and, in turn, those increased costs place additional strain on the resources needed to combat the rising tide of resistance (Figure 1).^1^

**Figure 1.**
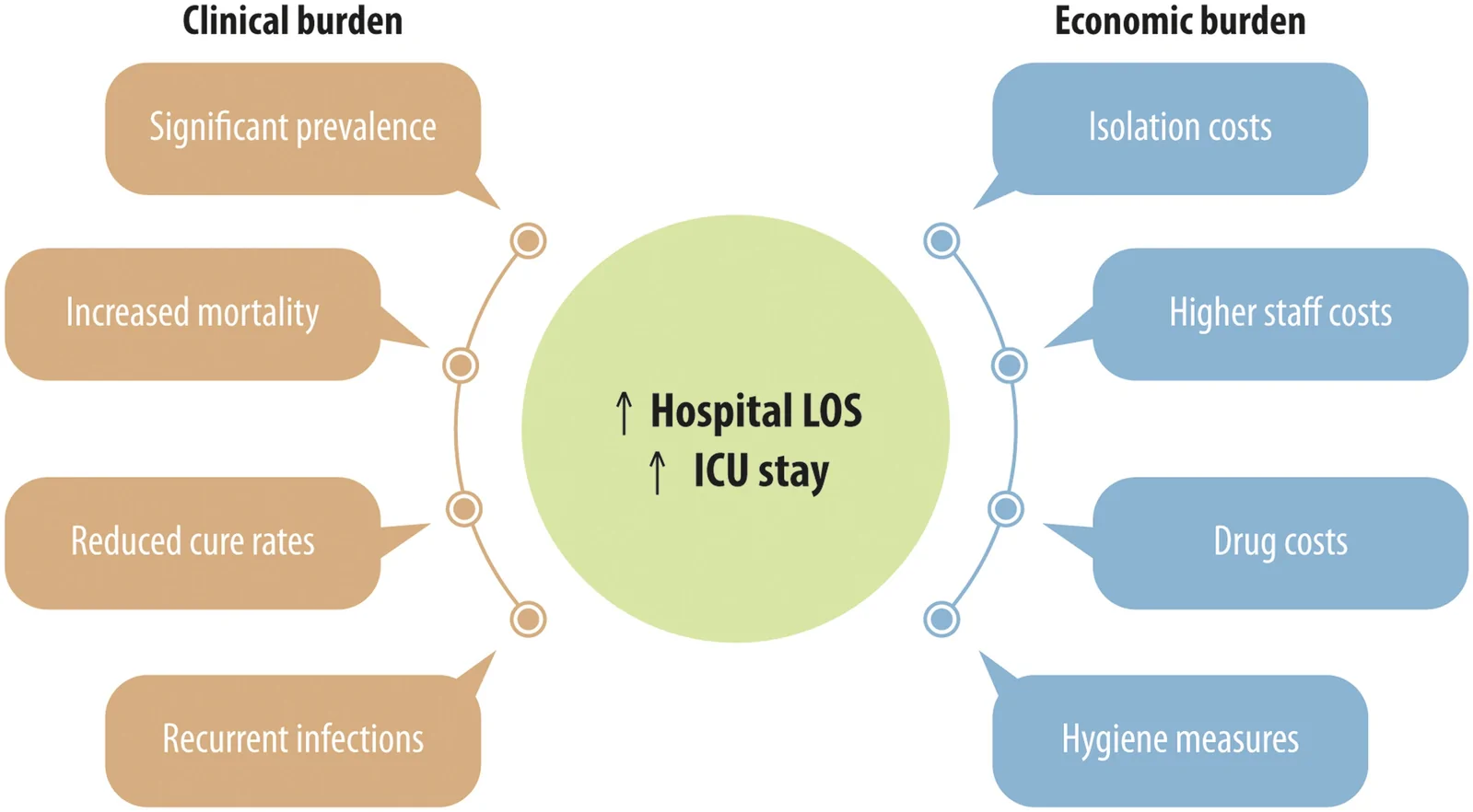
Clinical and economic burden of AMR. Modified from Eckmann and Tulkens.^1^ Licensed under the terms of the Creative Commons Attribution Licence (https://creativecommons.org/licenses/by/4.0/).

In 2014, the O’Neill report, commissioned by the UK Prime Minister, sounded a dire warning, projecting an alarming estimate of 10 million deaths attributable to AMR by 2050.^2^ Expanding on this, a 2022 study led by Naghavi analysed data from 204 countries and territories in 2019. The study reported an estimated 4.95 million deaths in 2019 associated with AMR in bacteria. This included 1.27 million deaths attributable to resistant bacteria, surpassing the death tolls of HIV/AIDS or malaria.^3^ The economic impact of AMR in Europe highlights substantial financial strains on healthcare systems, ranking as the third most significant threat to patients’ lives according to the Global Burden of Disease Collaborative Network.^4^ Gram-negative (GN) bacteria constitute the top five pathogens responsible for AMR and are associated with high morbidity and mortality rates.^3,5^ Additionally, multidrug-resistant Gram-negative bacteria (MDR-GN), characterized by their lack of susceptibility to at least one antibiotic in three or more antimicrobial categories, pose significant complications in clinical management, especially when it comes to choosing the correct initial treatment for critically ill patients (CIPs).^6^ MDR-GNs account for over one-third of all hospital-acquired infections (HAIs) in ICUs and are primarily attributed to carbapenem-resistant Enterobacterales (CRE), *Pseudomonas aeruginosa* and *Acinetobacter baumannii*.^7,8^ Their presence significantly increases mortality rates and imposes substantial financial burdens.^7,8^

Introducing new drugs or novel combinations has enhanced the treatment options, providing safer and more effective alternatives. However, defining and implementing the best clinical strategies for effectively dealing with these infections remains a critical need.^6^

Altogether, these data indicate the urgent need to consider evidence-based strategies that not only optimize the utilization of existing antibiotics but also focus significantly on the appropriate employment of new antimicrobial agents. A concerted and multidisciplinary effort is essential to ensure that these new therapeutic tools are and will be used effectively and sustainably, minimizing the risk of further resistance development and maximizing patient outcomes in complex clinical scenarios. Despite the availability of several new antibiotics and of guidance from international societies, a practical, coordinated antimicrobial stewardship framework specifically addressing how to enable timely access to these agents for CIPs with CRO infections—as opposed to managing MDR-GN infections in the general ward population—is still lacking. This review and the accompanying algorithm are intended to address that specific gap.

## Aims

The aim of this review is 4-fold:

1. Synthesize current research and guidelines, consolidating current research on the use of new antibiotics for CIPs, especially those with infections caused by CROs. This synthesis will address the shortcomings and challenges associated with traditional antibiotics.
2. Understand AMR dynamics in critical care with a special focus on the context of sepsis and septic shock.
3. Propose a balanced antimicrobial stewardship (AMS) strategy that reconciles the need to regulate and define the judicious use of antibiotics with the imperative to promptly deliver optimal therapy to CIPs with CROs.
4. Emphasize the role of a multidisciplinary approach in enhancing the effectiveness and minimizing the risks of new antibiotics within the framework of AMS.

This article adds the expert opinion of the authors to the reviewed evidence and is not intended to be simply an overview of existing literature.

## Methods

To support the objectives of this review, a focused literature search was conducted using PubMed, Scopus and Google Scholar, covering publications from January 2010 to July 2026, with a deliberate emphasis on studies published from 2020 onwards given the rapid evolution of the new-antibiotic landscape. Search terms combined controlled vocabulary and free-text keywords for three concept blocks: (i) the pathogens of interest (e.g. ‘carbapenem-resistant’, ‘multidrug-resistant Gram-negative’, ‘Enterobacterales’, ‘Pseudomonas aeruginosa’, ‘Acinetobacter baumannii’, ‘Stenotrophomonas maltophilia’); (ii) the antibiotics of interest, both by class and by individual agent name (e.g. ‘ceftazidime/avibactam’, ‘meropenem/vaborbactam’, ‘imipenem/relebactam’, ‘cefiderocol’, ‘ceftolozane/tazobactam’, ‘eravacycline’, ‘sulbactam/durlobactam’, ‘cefepime/enmetazobactam’, ‘aztreonam/avibactam’, ‘cefepime/taniborbactam’); and (iii) the clinical/critical-care context (‘critically ill’, ‘sepsis’, ‘septic shock’, ‘intensive care’, ‘antimicrobial stewardship’). These blocks were combined with Boolean operators, and reference lists of retrieved articles and recent guidelines were hand-searched for additional relevant citations (snowballing). Titles and abstracts were screened by the writing group for relevance to CIPs and CRO infections; full texts were retrieved when abstracts indicated original efficacy, safety, resistance, or stewardship data relevant to the review’s aims, and disagreements on inclusion were resolved by discussion among the authors. Because this is a narrative review intended to combine the available evidence with the authors’ collective clinical expertise, the search was not restricted to randomized controlled trials (RCTs) or systematic reviews; it also included observational studies, case series, editorials, commentaries and policy papers, which were used to illustrate real-world practice patterns, expert perspectives, and areas of ongoing uncertainty rather than to support quantitative efficacy claims. Additionally, websites of international organizations and societies (WHO, ECDC, IDSA, ESCMID) were consulted to ensure the review reflects the most current guidance available at the time of writing. No formal quality-assessment or risk-of-bias tool was applied, consistent with the narrative (rather than systematic) nature of this review; where evidence quality is particularly relevant to the strength of a statement, this is noted explicitly in the text.

## Early administration of antibiotics in septic shock

In scenarios involving sepsis and septic shock, it is critical to initiate prompt and effective antibiotic therapy as a pivotal measure to reduce mortality rates.^9–11^ Confirmation of infections in patients using standard microbiological methods can take up to 3 days, including time for antibiotic susceptibility testing (AST). During this period, authors agree that it is crucial to administer a broad-spectrum antibacterial treatment that covers the most likely pathogens.^12^ The complexity of treatment is exaggerated by the possibility of patients developing multiple infections with different pathogens at various times, complicating the assessment of treatment effectiveness for the initial infection.^12^ Yet, this approach needs to be carefully weighed against the hazards of administering antimicrobials unnecessarily; such risks include a range of adverse outcomes, such as renal impairment, thrombocytopenia, *Clostridioides difficile* infection, antibiotic-associated encephalopathy, allergic reaction and finally the rise of antimicrobial selective pressure.^13–16^ Notably, the mortality reduction linked to early antimicrobial use is most evident in septic shock cases, though this association is less pronounced in patients without septic shock, fuelling ongoing discussions about the ideal timing for initiating antibiotics in these cases.^11,13,17,18^

A recent multicentre cohort study on sepsis revealed that while antibiotic delivery within 1 h significantly correlates with lower in-hospital mortality in septic shock patients, this correlation is less distinct in non-shock sepsis cases. The study reported an adjusted OR for in-hospital mortality of 0.85 (95% CI, 0.64–1.15; *P* = 0.300) in the latter group.^17^ Furthermore, a retrospective analysis of over 104 000 adults, including septic shock and non-shock sepsis cases from 2015 to 2022, examined the impact of antibiotic timing on in-hospital mortality. While each hour's delay was linked to increased mortality in septic shock cases, significant impacts were only noted in non-shock sepsis cases after delays exceeding 6 h. This emphasizes the necessity of meticulous analysis in interpreting the relationship between antibiotic timing and mortality in sepsis.^19^ Despite this, several studies indicate that mortality risks may escalate after 3–5 h of delay post-hospital arrival or sepsis recognition.^13,20–22^ Consequently, the 2026 Surviving Sepsis Campaign recommends initiating antibiotics in patients with potential sepsis without shock as soon as sepsis is deemed the most likely diagnosis and certainly no later than 3 h following the initial suspicion of sepsis if concerns remain.^9^ It is important to note that the studies underpinning these time-to-antibiotic recommendations were conducted predominantly in unselected septic populations and did not stratify patients by risk of infection with MDR organisms or CROs; consequently, these timing targets cannot be directly extrapolated to guide the specific timing of new, targeted antibiotics in CIPs at high risk of CRO infection, for whom the diagnostic and therapeutic pathway discussed (Figure 2) is more directly relevant.^13,20–22^

**Figure 2.**
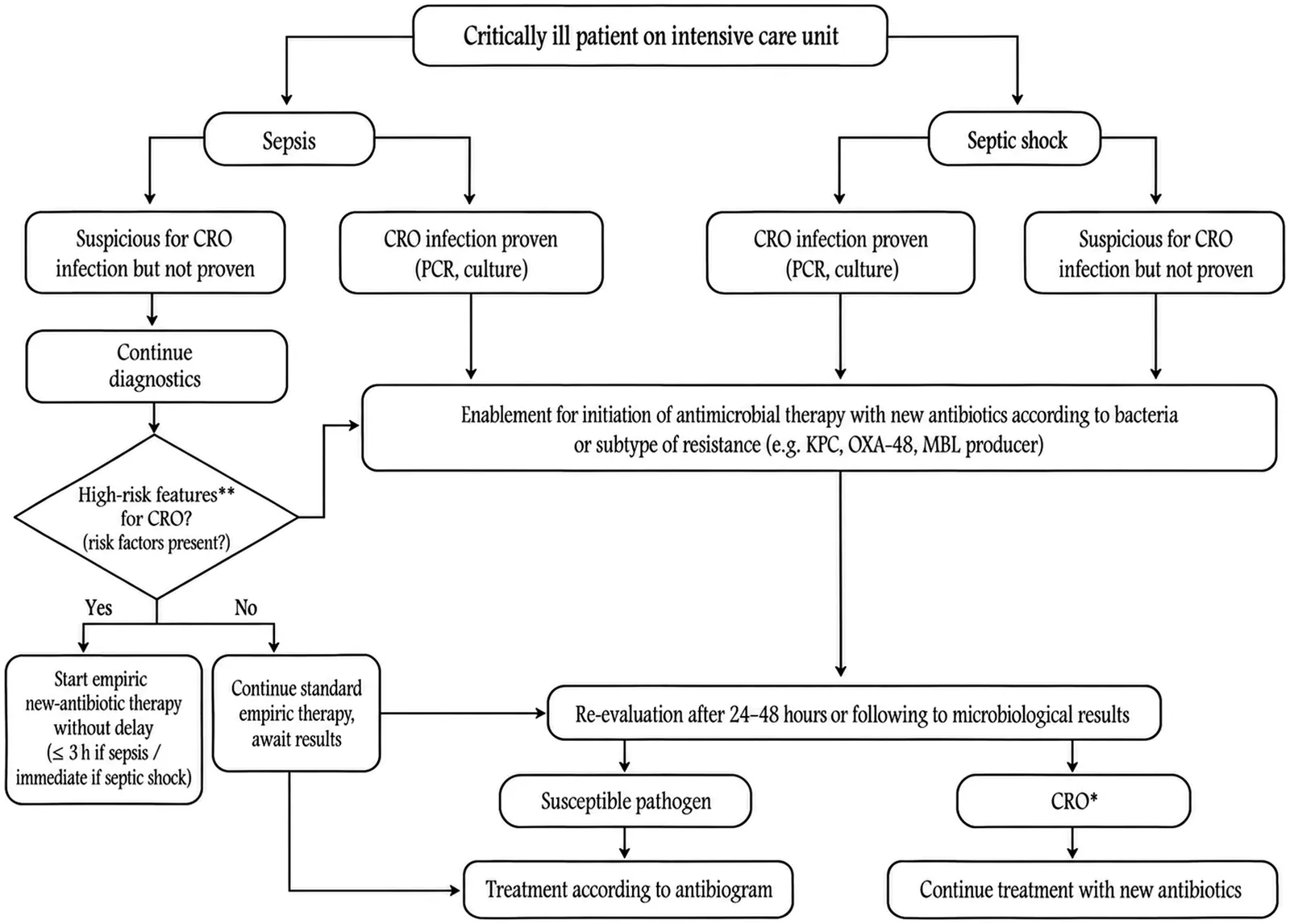
Algorithm for treatment of septic ICU patients suffering from CRO infections. *In case of *S. maltophilia* or other non-fermenting Gram-negative bacteria the treatment should be individualized. **High-risk features may include prior colonization/infection with a CRO, high local prevalence of CRO, recent exposure to broad-spectrum antibiotics, and septic shock at presentation. CRO, carbapenem-resistant organism; KPC, *K. pneumoniae* carbapenemase; MBL, metallo-beta lactamase; OXA-48, oxacillinase-48; PCR: polymerase chain reaction.

Administering antibiotics early leads to improved outcomes in CIPs. A retrospective study involving over 17 000 ICU patients with sepsis or septic shock revealed a direct link between mortality and delays in the initial antibiotic treatment.^23^ Another study involving 35 000 septic patients demonstrated that each hour of delay in antibiotic administration after registration in the hospital was associated with a 9% increase in mortality risk.^18^ The mortality risk progressively increased with delays in treatment initiation, reaching 37% within 48 h, and 57% within 72 h.^24^ A recent study also investigated the correlation between the timing of treatment initiation and outcomes in over 19 000 paediatric patients with suspected sepsis in US hospitals. Patients who received antibiotic treatment within 5.5 h from hospital admission presented a reduced 3 day and 30 day mortality compared with those who received treatment after this time frame (0.5% and 0.9% versus 1.2% and 2%, respectively).^25^

Another crucial aspect is the duration of antibiotic treatment, as a shorter antibiotic course may reduce selective pressure, limiting the emergence of resistant pathogens.^26,27^ A recent meta-analysis compared the outcomes of 7 day and 14 day treatments in more than 1000 patients hospitalized with GN bacteraemia and showed that both treatments presented similar mortality rates and related outcomes (30-day mortality of 4.4% and 4.1%, respectively).^28^ A study comparing short- and long-course antibiotic treatments for Enterobacterales-related bacteraemia showed that 4.4% of MDR-GN resistance was reported following short treatment, while 7.3% was registered following long treatment.^29^ Thus, the prompt initiation of antibiotics, preferably within 24 h of hospitalization or the onset of sepsis, along with short-course antibiotic treatment strategies, constitutes the most effective approach to achieve a balance between health-related outcomes and minimizing the emergence of antibiotic resistance.^26,30^

On a global scale, the economic impact of sepsis and septic shock, particularly when involving MDR pathogens, is substantial. This is evident in the cost contrast between treating abdominal sepsis with and without MDR involvement; the former incurs €40 000 per patient versus €27 000, as per the study conducted by De Pascale *et al.*^31^

## The medical and economic burden of infections due to MDR-GN

As previously anticipated, research indicates that infections caused by MDR-GN are important risk factors correlated with higher mortality rates compared with infections caused by susceptible strains in CIPs and ICUs.^32–34^

In a prospective study conducted across nine hospitals in Italy from December 2010 to May 2011, researchers focused on patients with carbapenem-resistant *K. pneumoniae* (CR-KP) isolations. The study found that the overall in-hospital mortality rate for patients infected with CR-KP was 25.8%. Notably, the mortality rates differed significantly between patients with infections caused by colistin-susceptible KP (20.3%) and those with colistin-resistant CR-KP (40.6%, *P* = 0.04). Furthermore, infection with a colistin-resistant strain emerged as an independent risk factor for in-hospital mortality (OR = 4.15; 95% CI, 1.17–14.74; *P* = 0.02).^32^ Another study examining the prognostic significance of *Klebsiella* spp. in the ICU found that mortality rates for infections caused by CR-KP were 20% higher than those associated with infections caused by carbapenem-susceptible strains. This underscores the correlation between the presence of resistant microorganisms, whether in the form of colonization or infectious agents, and increased mortality.^35^ In a comprehensive observational multicentre study, AMR in GN bacteria to include ESBL production or carbapenem resistance was significantly linked to higher mortality rates.^33^

A prospective multicentre study from Italy demonstrated that bloodstream infections (BSIs) caused by CR-GN bacteria are associated with a significant excess of mortality compared with those caused by carbapenem-susceptible (CS) strains, and the major impact was recorded in patients infected by MBL-producing Enterobacterales, followed by *P. aeruginosa* and *A. baumannii.*^36^ These data emphasize the need for patient-specific risk stratification when dealing with such infections to balance the necessity of adequate empirical treatment against the risk of resistance selection. This approach is particularly important when an MDR-GN pathogen is suspected.^37^ Patients with more risk factors, such as older age and comorbid conditions, are more likely to be infected by MDR pathogens.^33^ On the other hand, in ICU settings, patients infected with MDR pathogens often have treatment limitations, which may lead to increased mortality, less stringent antibiotic stewardship, and weaker implementation of infection prevention methods.^33^

## Tailoring antimicrobial therapy in CIPs suffering from MDR-GN infections

In managing CIPs, the decision to use narrow- or broad-spectrum antibiotics should be founded on a thorough understanding of the pathogen's resistance profile (also based on local epidemiology) and essential pharmacokinetic/pharmacodynamic (PK/PD) factors, such as tissue penetration and safety. Furthermore, while the ideal scenario involves tailoring antimicrobial therapy based on specific pathogen identification and susceptibility tests, clinical realities often necessitate the rapid use of empirical therapy, particularly in severe infections.^13,38^

A multidimensional approach is highly recommended when considering empirical antibiotic treatment for MDR-GN pathogens. This approach should consider the local epidemiology of MDR-GN pathogens, patient risk factors, the site of infection, and the severity of the illness, including mortality risk.^7,39,40^

This is especially crucial in countries with a high prevalence of resistant pathogens. A study from Italy showed that in an endemic area up to 33% of patients with rectal colonization by CRE were diagnosed at hospital admission, and the risk of developing secondary BSI was higher in patients with multisite extraintestinal colonization, intravascular device, solid organ transplant, undergoing invasive procedures, admitted to ICU, receiving cephalosporins or fluoroquinolones, and with colonization by NDM-producing *K. pneumoniae*.^41^ Notwithstanding the significant prevalence of CR-GNB, a study conducted among non-infectious disease specialists in Thailand revealed that empirical antibiotic treatments were generally not administered owing to the absence of standardized guidelines and requisite information.^42^

Significant risk factors for MDR-GNs include antibiotic exposure in the previous 90 days and prior infection or colonization with MDR-GNs in the past 12 months.^43^ Also, empirical coverage for MDR-GN pathogens has been recommended if the hospital prevalence shows more than 20% resistance to a potential empirical agent.^40^ AST should be performed to guide empirical therapy; however, this might not be available promptly for newer antimicrobials as many laboratories do not report such susceptibility unless requested by the clinician.^39^

After selecting an empirical regimen, antimicrobials should be dose-optimized, considering patient and microbiological data for the targeted pathogens. It is imperative to closely monitor diagnostic testing outcomes, including Gram stain results, traditional cultures and rapid diagnostic test (RDT) findings. This continuous review is essential for identifying opportunities to either escalate or de-escalate antimicrobial therapy, aligning it more precisely with the targeted or identified pathogen.^39,43,44^

## Strengths and weaknesses of de-escalation

Antimicrobial de-escalation (ADE) is a strategic strategy within AMS aimed at curbing the emergence of AMR by reducing the use of broad-spectrum antimicrobials when not needed. However, its definition is subject to variations, leaving a gap in evidence-based guidelines for its application in ICUs. In a recent review based on the Delphi method, a group of 16 experts reported that ADE in CIPs may involve several strategies. Firstly, it can mean replacing broad-spectrum antimicrobials with narrower-spectrum agents or those with a lower ecological impact, which is not always the same. It could also involve discontinuing antibiotic combination therapy. It is important to note that simply discontinuing all antimicrobial therapy when an infection is ruled out is not considered by the authors as a form of ADE.^44^

In terms of the effects of ADE, it is considered likely safe for patients’ outcomes, such as mortality and LOS in the hospital. However, there is a risk that ADE could increase the total duration of antimicrobial therapy. Therefore, ADE and the duration of therapy should be considered separately but as part of an overall AMS strategy.^44^ The safety of ADE has been established through research. Numerous observational studies have found that implementing ADE does not lead to an increase in mortality, duration of treatment or LOS.^45^

## PK/PD considerations and therapeutic drug monitoring

A multidimensional approach is especially important in CIPs, considering that these patients often experience abnormal volume distribution, haemodynamic fluctuations, and renal or liver dysfunction. In this sense, PK/PD principles and therapeutic drug monitoring (TDM) underpin the dosing and monitoring recommendations for the new antibiotics and constitute a base for their responsible use in critically ill patients. Conducting specific PK/PD studies for different CIP groups is crucial to enhance antibiotic effectiveness in treating deep-seated infections and to assess the efficacy of combination therapies in combating resistance. In the ICU setting, the fluctuation of renal function necessitates tailored dose adjustments of antibiotics guided by validated creatinine clearance (CrCl) formulas. This may facilitate managing increased renal clearance and avoiding premature dose reductions during the initial treatment phase.^7,46^ Additionally, it is vital to monitor the achievement of PK/PD targets in patients undergoing renal replacement therapy (RRT).^46,47^ Factors such as capillary leakage, fluid therapy and hypoalbuminaemia can significantly increase the volume of distribution (*V*) for antibiotics. This expansion leads to the dilution of antibiotics in body fluids, resulting in variable drug levels.^46,47^ Moreover, physiological changes arising from conditions like trauma, burns or the administration of haemodynamically active drugs can influence the renal clearance of antibiotics.^6,46^ The use of extracorporeal support techniques further complicates antibiotic dosing. PK studies in septic ICU patients have shown significant daily variations in drug levels and clearance, which correlate with changes in CrCl.^46,47^

It was suggested that the optimal bactericidal effects are obtained when the antibiotic concentration is 4-fold above the MIC for 40%–60% of the dosing interval.^48^ Moreover, β-lactam antibiotics typically have a time-dependent activity. A meta-analysis including 18 RCTs and 13 non-RCTs revealed that a continuous infusion strategy could improve clinical cure and reduce hospitalization stays and mortality in CIPs, compared with intermittent strategies.^48^ Additionally, loading doses, generally considered the fundamental treatment doses, produced superior improvements in CIPs compared with patients who did not receive them.^48,49^

The adoption of bedside TDM could further individualize treatment, and its integration into standard care practices is advocated, although not yet fully available.^6,7^ TDM implementation is affected by economic disparities in accessing MIC values among countries. Limitations of drug availability and delayed diagnostic results represent the most critical barriers to the global implementation of TDM.^50^

## Issues of ‘old’ antibiotics in infections due to CRO

Amongst MDR-GN, carbapenem-resistant *A. baumannii* (CRAB), carbapenem-resistant *P. aeruginosa* (CR-PA) and CRE have been classified as critical priority pathogens by the WHO.^51^ Moreover, since 2019, the CDC has identified CRE and CRAB as two of the most urgent threats.^52^ In the USA alone, CRE account for approximately 1100 deaths and 13 100 infections annually.^51^ A substantial proportion of these infections lead to fatalities, largely due to the limited options available in antibiotic treatments.^53^

Adding to this concern, the ECDC reported in 2019 a rising prevalence of CRE across Europe.^54^ Notably, 43% of European countries indicated the regional or inter-regional spread of CRE. In 2017, surveillance data showed 137 728 cases of carbapenem-resistant *E. coli* and 32 461 cases of CR-KP, highlighting their growing impact on European healthcare systems.^55^

CRO infections are associated with high mortality rates, especially in neonates, children and several patient populations, including immunocompromised patients, those with pancreatic diseases, transplant recipients, patients who require devices like ventilators, urinary or IV catheters, and patients in ICUs.^56–61^

Research has shown that patients colonized with CRO have about a 1.79 times higher risk of mortality in ICU compared with those without colonization.^62^ Closely linked to these issues, the economic burden of treating CRO colonization is substantial. These infections often require more extended hospital stays, more complex and expensive treatment regimens, and additional care due to complications. For example, in a study conducted over 2 years at the Central London Hospital, it was found that the median healthcare-related cost of treating a patient with a CRO infection was significantly higher, more than double (£49 537), compared with the cost of treating a patient with a carbapenem-sensitive organism (CSO), which was £19 299. The greatest portion of these costs was attributed to critical care. This study also revealed often overlooked expenses, such as patient support, portering and catering. Furthermore, patients with CRO infections experienced longer hospital stays and increased time in the operating theatre than those with CSO infections.^63,64^

The efficacy of existing antibiotics against CROs is increasingly questioned. Despite reintroducing older antibiotics like colistin and polymyxin B, their effectiveness is often limited. This limitation is attributed to various factors, including the development of resistance and the PK/PD properties of these drugs.

Colistin (polymyxin E) and polymyxin B, introduced in the 1950s, have re-emerged as critical agents in treating CRE and other CRO infections. Their resurgence as salvage therapy for otherwise untreatable GN infections comes despite not having undergone modern drug development processes. This has led to challenges in their clinical application, including confusion over dosing conventions, formulation differences, outdated product information, and uncertainties in susceptibility testing, complicating their optimal use.^65^

These antibiotics, differing by only a single amino acid, belong to the last-resort agents against CRE infections. However, they present significant toxicity challenges, particularly to the CNS and kidneys. In the past, neurotoxicity was a concern when using polymyxins, but with the formulations used today, this adverse effect is less commonly reported. This could also be because the recent literature often involves patients who are more critically ill, under ventilation and sedation. These factors may significantly impede the detection of neurotoxicity, primarily identified by symptoms like paraesthesias and ataxia. On the other hand, nephrotoxicity remains a significant issue, as it still occurs in more than 40% of patients treated with polymyxins. Reports indicate that nephrotoxicity is observed with both colistin and polymyxin B, but recent studies suggest a higher incidence of nephrotoxicity with colistin (50%–60%) compared with polymyxin B (20%–40%).^66^ For example, a colistin dose of 5 mg/kg body weight/day was independently predictive of the development of renal insufficiency in a retrospective analysis.^67^

A further issue related to the use of polymyxins is the development of resistance during treatment. In one case, blood samples from a patient with a CR-KP infection undergoing treatment with polymyxin B monotherapy displayed a marked increase in the polymyxin B MIC within just 5 days, rising from 0.75 mg/L to 1024 mg/L.^68^ Moreover, there have been instances of outbreaks involving colistin-resistant, carbapenem-resistant *K. pneumoniae*.^69,70^

Tigecycline, a glycylcycline antibiotic, showed initial promise in treating resistant infections, including those from CROs.^71^ Despite its broad-spectrum activity against MDR pathogens, its clinical efficacy, particularly for BSIs and pneumonia caused by CROs, is limited partly due to its PK profile, leading to lower serum and lung tissue concentrations.^72^ This limitation hampers its effectiveness in critical infection sites. Moreover, the increasing emergence of tigecycline-resistant strains has further limited its utility, emphasizing the need for alternative or combination therapies to manage CRO infections effectively. In 2010, the FDA warned of higher mortality risks with tigecycline in severe infections.^73,74^ However, its ability to counter common tetracycline resistance mechanisms makes it a potential option, especially against rising drug resistance in CRE, for which high doses of tigecycline are considered an alternative option for treatment of complicated intra-abdominal infection (cIAI), and skin and soft tissue infections (SSTIs) when β-lactam agents cannot be used. Still, due to the high mortality rates, particularly with monotherapy, combining tigecycline with other antibiotics might be essential for better treatment outcomes.

## Recent data on new antibiotics in infections due to CRO

The introduction of new treatment options involving either newly developed or existing β-lactam molecules combined with innovative or traditional β-lactamase inhibitors has provided clinicians with enhanced options to effectively manage infections caused by MDR-GN bacteria (MDR-GNB).

Most of the data on the use of new antibiotics in MDR-GNB infections derive from small observational studies. At the same time, large clinical trials include only a small percentage of this category of microorganisms.

Recruiting eligible patients for RCTs on AMR infections has largely failed due to various barriers.^75^ The main factors limiting recruitment stem from the severe conditions of eligible patients and timely interventions. Furthermore, recruitment regulations can differ globally, making enrolling patients from various locations challenging.^76^ It is also crucial that treatment begins on the first day/days after diagnosis, and rapid diagnostic tests are unavailable in several settings; many eligible patients are excluded from RCTs due to the inability to follow the RCT protocol promptly.^76^ High costs also serve as a barrier. Developing new antibiotics requires substantial investments, and the return is uncertain due to low sales volumes at modest prices. This may create a vicious cycle since regulatory approval for new antibiotics relies on non-inferiority clinical trials, and recruiting for these trials is difficult.^77^ New strategies are being proposed to improve patient recruitment, mostly based on eligibility criteria, trial designs and strategies to obtain informed consent from CIPs.^76^ However, the need for real-world experiences is still elevated.

To support the daily practice of clinicians, the IDSA and ESCMID have published specific recommendations. The IDSA guidance was first published in January 2022 and subsequently updated based on new data until 2026; it provides recommendations based on a non-systematic review of literature coupled with the clinical experience of the panelists.^43^ The ESCMID guidelines were published in the spring of 2022 and no updates have been published to date^78^; they are developed through a systematic literature review and classification of evidence according to GRADE methodology; therefore, they tend to be more cautious and conservative than IDSA guidance on many issues.^43,78^

Table 1 briefly compares first-line antibiotics suggested for MDR-GN severe infections by the two guidelines. Concerning CRO infections, there are several discrepancies between the two documents; e.g. differently from the IDSA, the ESCMID guidelines are neither for nor against the use of imipenem/relebactam in CRE infections, in the absence of sufficient evidence.^43,78^ Similar conclusions are reported for cefiderocol, imipenem/relebactam and ceftazidime/avibactam in CR-PA infections. Moreover, based on the results of CREDIBLE and APEKS-NP trials, the ESCMID guidelines recommended against using cefiderocol to treat infection caused by carbapenem-resistant *A. baumannii* (CRAB), although on low-certainty evidence.^78^

**Table 1.** IDSA and ESCMID recommendations on first-choice antibiotic regimen for infections caused by carbapenem-resistant Gram-negative bacteria assuming *in vitro* susceptibility, unless otherwise specified

CR Enterobacterales						CR *P. aeruginosa*			CR *A. baumannii*	*S. maltophilia*
Infection site	cUTI	Others outside urinary tract				cUTI	Others outside urinary tract	All	All	All
Carbapenemase production test	Not specified	Not available or negative	KPC	OXA-48	MBL	Not specified		MBL	Not specified	Not specified
IDSA 2026	SXT, CIP, LVX, CZA, MVB, IMR, FDC, ATM-AVI	MVB, IMP^a^	MVB, CZA, IMR Alternative: ATM-AVI, FDC	CZA Alternative: FDC, ATM-AVI	ATM-AVI, FDC Alternative: CZA + ATM	C/T, CZA, IMR, FDC	C/T, CZA, IMR, Alternative:FDC	FDC	SUL/DUR + carbapenem (MEM/IMP) Bridge therapy only: HD SAM^b^ + PMB, MIN/TGC15, or FDC, until SUL/DUR available	FDC monotherapy Alternative: ATM-AVI + 2nd agent, or 2 of: SXT, MIN, LVX
ESCMID 2022	MVB, CZA				FDC, CZA + ATM	C/T			SAM^c^, (PMB, HD TGC15)	Not addressed

ATM, aztreonam; C/T, ceftolozane/tazobactam; CIP, ciprofloxacin; CR, carbapenem-resistant; cUTI, complicated urinary tract infection; CZA, ceftazidime/avibactam; FDC, cefiderocol; HD, high-dose; IMP, imipenem; IMR, imipenem/cilastatin/relebactam; KPC, *Klebsiella pneumoniae* carbapenemase; LVX, levofloxacin; MEM, meropenem; MIN, minocycline; MVB, meropenem/vaborbactam; PMB, polymyxin B; SAM, ampicillin/sulbactam; SUL/DUR, sulbactam/durlobactam; SXT, trimethoprim/sulfamethoxazole; TGC15, tigecycline.

^a^If risk factors for MBL-producing bacteria are present: CZA + ATM, FDC.

^b^Irrespective of *in vitro* susceptibility.

^c^Only if confirmed *in vitro* susceptibility.

### Ceftazidime/avibactam

Ceftazidime/avibactam is a combination of a third-generation cephalosporin, ceftazidime, with a new non–β-lactamic β-lactamase inhibitor, avibactam. Ceftazidime/avibactam is active *in vitro* against Ambler class A (*K. pneumoniae* carbapenemase, KPC) and certain class D (e.g. OXA-48) carbapenemases but is inactive against MBL producers.^79^

In the EU, ceftazidime/avibactam is approved for the treatment of adults and children older than 3 months with cIAI, complicated urinary tract infection (cUTI), hospital-acquired pneumonia (HAP), including ventilator-associated pneumonia (VAP), and BSI associated with these infection sources. It is also licensed for other infections caused by aerobic GNB in patients with limited treatment options.^80^

Evidence for recommending the use of ceftazidime/avibactam over other antibiotics for the treatment of CRO infections is based only on observational real-life studies. More recently, a systematic review with meta-analysis, including 11 studies and 1200 patients with BSI caused by CRE, supported the evidence from real-life experiences over the past 10 years, showing better clinical outcomes in ceftazidime/avibactam-treated patients compared with those treated with conventional therapy.^81^ Ceftazidime/avibactam showed optimal results also in the treatment of infection caused by OXA-48 producers in three comparative studies.^82–84^

Regarding the treatment of infections caused by MBL-producing bacteria, an association of ceftazidime/avibactam with aztreonam has shown a synergistic effect *in vivo*. An observational study including patients with BSI caused by MBL-producing CRE (mostly *K. pneumoniae* NDM-producers) reported a 30-day mortality rate significantly lower in patients treated with this combination than other regimens even when colistin was included (19.2% versus 44%, *P* = 0.007).^85^

A meta-analysis of 11 observational studies on CR-GNB infections, including difficult-to-treat (DTR) *P. aeruginosa* (DTR-PA), found no differences in mortality or microbiological cure between ceftazidime/avibactam alone and combination therapy. However, the analysis was limited by only 19 monomicrobial cases of DTR-PA.^86^ A retrospective monocentre study conducted in China in 136 patients with CR-PA infections revealed better clinical outcomes in patients treated with ceftazidime/avibactam compared with patients receiving polymyxin B.^87^ Treatment-emergent resistance to ceftazidime/avibactam has been described but the incidence remains low and heterogeneously reported.^88^

### Meropenem/vaborbactam

Vaborbactam is a novel cyclic boronic acid inhibitor that restores meropenem activity against producers of numerous class A and C β-lactamases, but it is inactive against MBL and OXA-48 producers.^89^ In the EU, meropenem/vaborbactam is approved for the treatment of adults with cIAI, cUTI, HAP/VAP and BSI secondary to these infection sources; it is also licensed for other infections caused by aerobic GNB in patients with limited treatment options.^90^

RCTs investigating the use of meropenem/vaborbactam against CRO are limited. In TANGO I, a Phase III, multicentre, double-blind RCT, comparing meropenem/vaborbactam with piperacillin/tazobactam in treating cUTI, the occurrence of CRE was limited.^91^ In TANGO II, a randomized, multicentre, open-label Phase III study, patients with cUTI, cIAI, HAP and BSI caused by CRE were enrolled. Meropenem/vaborbactam was compared with physicians’ choice of the best available antibiotic treatment (BAT), also including monotherapy with ceftazidime/avibactam. The final analysis included 47 patients; significantly improved cure rates at the end of treatment were found in the meropenem/vaborbactam group (65.6% versus 33.3%, *P* = 0.03) with non-significantly lower 28 day mortality (15.6% versus 33.3%, *P* = 0.2) and lower rates of renal-related adverse events.^92^

Alosaimy *et al.*^93^ conducted a retrospective cohort study with 126 patients having suspected or confirmed infections from MDR-GNB, where CRE comprised 78.6% of cases. Most infections originated in the respiratory tract (38.1%) and intra-abdominal region (19%). According to a multivariable analysis, early treatment with meropenem/vaborbactam was independently linked to more favourable clinical outcomes, with an adjusted OR of 0.277 (95% CI, 0.081–0.941). In a retrospective observational cohort study conducted at 12 Italian hospitals, 37 patients with infections attributed to KPC-producing *K. pneumoniae* (KPC-Kp) were assessed; 70% of these cases were identified in ICUs. Among the 37 cases, clinical cure was achieved in 28, but 3 of these patients, all of whom had BSIs, experienced a recurrence after discontinuing meropenem/vaborbactam treatment. Importantly, the isolated bacteria remained susceptible to meropenem/vaborbactam.^94^

The efficacy of meropenem/vaborbactam has been directly compared with ceftazidime/avibactam in one multicentre, retrospective cohort study. This work focused on infections due to KPC-producing Enterobacterales (72% of cases) and roughly half of participants were CIPs. The two drugs showed similar results in terms of clinical and microbiological successes, LOS, incidence of adverse events and mortality.^95^

### Imipenem/cilastatin/relebactam

Relebactam is a new β-lactamase inhibitor that produces a dose-dependent synergy with imipenem against CRE-producing KPC, but it is poorly active against OXA-48 and MBL producers; moreover, it protects imipenem against degradation by *P. aeruginosa*’s cephalosporinase.^96^

Cilastatin functions as an inhibitor of renal dehydropeptidase. It does not possess antibacterial properties itself but plays a crucial role in preserving the serum concentrations of imipenem. It achieves this by inhibiting the renal metabolism of imipenem. Additionally, cilastatin is believed to offer nephroprotective benefits.^97^ Imipenem/relebactam is approved in the EU for treating adults with HAP/VAP and related BSI, and for infections caused by aerobic GNB when other treatments might be ineffective.^98^ In the RESTORE-IMI 1 study, a double-blind, Phase 3 RCT, imipenem/relebactam was found to have comparable efficacy and better safety than the combination of imipenem/cilastatin plus colistin in patients with cUTI, cIAI and HAP/VAP. Among 31 patients with CR-GNB included in the modified microbiological intention-to-treat (mMITT) population, 24 (77.4%) had infections caused by difficult-to-treat resistant *P. aeruginosa* (DTR-PA) and only 7 by CRE.^99^ Other clinical trials of imipenem/relebactam, like RESTORE-IMI 2, included only few or no patients with CRO, so that meaningful analyses cannot be performed.^100,101^ A recent multicentre study presented real-world observations of imipenem/relebactam usage and indicated that it is primarily used to treat multidrug-resistant (MDR) *P. aeruginosa* and, in some instances, CRE. A notable outcome is that imipenem/relebactam led to clinical cure in most cases, despite a mortality rate of 33%.^102^ It is important to note that patients treated with imipenem/relebactam typically have high APACHE II scores, which are associated with mortality rates of around 40%, which are higher compared with those in the RESTORE-IMI 1 trial.^102^

In a recent meta-analysis, the authors conducted a comprehensive review and evaluation of RCTs and observational studies, focusing on the efficacy and safety of imipenem/relebactam. This combination was compared with alternative antibiotics for treating cUTIs, cIAIs, HAP and VAP. Data indicated the non-inferiority of imipenem/relebactam compared with other treatments such as imipenem, piperacillin/tazobactam, and imipenem combined with colistin, particularly for the treatment of CR-GN infection, suggesting that imipenem/relebactam could be a viable alternative for treating serious bacterial infections that are increasingly resistant to carbapenems.^103^

### Cefiderocol

Cefiderocol is a siderophore cephalosporin that is relatively stable against hydrolysis by most serine and MBL (including KPC, NDM, VIM, IMP and OXA) carbapenemases.^104^

Cefiderocol is indicated for the treatment of infections due to aerobic GNB in adults with limited treatment options.^105^ Most of the data about the clinical efficacy of cefiderocol derived from three RCTs: the APEKS-cUTI, APEKS-NP and CREDIBLE-CR studies. The APEKS-cUTI and APEKS-NP studies compared cefiderocol with carbapenems in cUTI and nosocomial pneumonia, respectively, both resulting in non-inferiority of cefiderocol. Among isolates, nonetheless, CROs were relatively underrepresented.^106,107^ Most of the data about the clinical efficacy of cefiderocol in CROs were derived from the CREDIBLE-CR study. In this open-label, multicentre, pathogen-focused, descriptive, Phase III trial, patients with HAP, VAP, HCAP, cUTI and BSI caused by CR-GNB were randomized to receive either cefiderocol or BAT, mostly based on colistin or ceftazidime/avibactam combination regimen. Cefiderocol had similar clinical and microbiological efficacy to BAT. In patients with infections caused by CRE, 19 out of 29 (66%) in the cefiderocol group and 5 patients out of 11 (45%) in the BAT group achieved a clinical cure. In patients with infections caused by organisms producing MBLs, clinical cure was achieved by 12 of 16 (75%) with cefiderocol and 2 of 7 (29%) with BAT. More patients died in the cefiderocol group than in the BAT group by the end of the study: 34 (34%) of 101 versus 9 (18%) of 49. This difference was mainly noted in patients with severe infections caused by *Acinetobacter* spp. There was no mortality difference among patients with CRE infections.^108^

Ten patients with OXA-48-producing isolates were enrolled across the APEKS-NP and CREDIBLE-CR studies. Seven of these patients achieved clinical cure, one experienced clinical failure, and two had indeterminate outcomes.^109^ The use of cefiderocol treatment resulted in the eradication of MBL-expressing CRE in 7 out of 10 patients by the end of the treatment period. Additionally, in the CREDIBLE-CR study, 8 out of 10 patients achieved clinical cure at the test-of-cure stage. In the APEKS-NP study, 60% of the MBL-producing CRE infections were successfully eradicated, and 60% of the treated patients achieved clinical cure.^110^ Similar results were found in a case series of 18 Italian patients.^111^ However, in a real-world, multicentre study in Italy (CEFI-BAC), the presence of NDM-producing *K. pneumoniae* isolates was an independent predictor of 30 day mortality in patients treated with cefiderocol.^112^

Two retrospective Italian studies explored the role of cefiderocol in treating severe CRAB infections, BSI and HAP/VAP. In a retrospective study conducted at a single centre with 124 patients, the authors found that 30-day mortality was significantly higher in those treated with colistin compared with those receiving a cefiderocol-containing regimen (55.8% versus 34%, *P* = 0.018). This discrepancy was confirmed in the subgroup of patients exhibiting BSIs; however, it was not observed in those with VAP. Nevertheless, microbiological failure occurred in 17.4% of the cefiderocol group versus 6.8% of the colistin group.^113^ Another retrospective multicentre study did not find any statistically significant differences in all-cause 28 day mortality between patients treated with cefiderocol compared with those receiving alternative regimens (55% versus 58%, *P* = 0.7), and in the multivariate analysis cefiderocol was associated with non-significant lower mortality risk (HR 0.64; 95% CI, 0.38–1.08, *P* = 0.10).^114^

Recently, a rise in cefiderocol resistance has been observed. In a recent case series from Italy, three isolated strains of KPC-KP exhibited co-resistance to aztreonam/avibactam and cefiderocol following treatment, due to a deletion in the carbapenemase gene.^115^ Mutations in genes that encode siderophores have also been identified in CR-KP isolates exhibiting a cefiderocol resistance phenotype developed after 32 days of cefiderocol therapy.^116^ Of note, new clinical trials are being conducted combining cefiderocol and xeruborbactam, a cyclic boronate inhibitor that inhibits serine- and MBL carbapenemases.^117^

### Ceftolozane/tazobactam

Ceftolozane inhibits PBPs and exhibits activity on isolates habouring non-ESBL TEM and SHV variants and AmpC enzymes, while tazobactam targets class A serine-lactamases and ESBLs. The combination of ceftolozane/tazobactam lacks activity against carbapenemases and has partial activity on AmpC β-lactamases.^118^ Conversely, it is one of the most active antipseudomonal agents; its activity against *P. aeruginosa* exceeds the rest of the antipseudomonal lactams by 20%–25%.^119^

Ceftolozane/tazobactam is approved in the EU for treatment of adults with cIAI, cUTI including acute pyelonephritis, and HAP/VAP.^120^ Registration trials have established the drug's non-inferiority to meropenem and levofloxacin in cIAI and HAP/VAP and in cUTI, respectively, confirming *in vivo* efficacy against ESBL-producing Enterobacterales.^121–123^ As with other new agents discussed here, these registration trials enrolled largely non–critically ill populations and did not specifically evaluate outcomes in septic shock, limiting direct extrapolation to CIPs. Some case series of clinical experiences and one retrospective study on 205 patients treated with ceftolozane/tazobactam for MDR/XDR *P. aeruginosa* infections have been published. The main indication in these series was respiratory tract infections (RTIs), and different doses of ceftolozane/tazobactam were used, sometimes in combination therapy. Cure rates were close to 70%, with emergence of resistance of 4% to 14% in some studies.^124–128^

A systematic review and meta-analysis evaluating clinical efficacy and safety of ceftolozane/tazobactam in treating GNB infections in adults has been published. A total of 13 studies were included in the analysis. Patients treated with ceftolozane/tazobactam demonstrated significant advantages in terms of clinical cure (OR 1.62; 95% CI, 1.05–2.51) and microbiological eradication (OR 1.43; 95% CI, 1.19–1.71), particularly in cases of *P. aeruginosa* infections. When compared with polymyxin/aminoglycoside or levofloxacin, ceftolozane/tazobactam also showed a clear advantage in terms of clinical success and microbial eradication. There were no notable differences in adverse events, *Clostridioides difficile* infection, or mortality between patients treated with ceftolozane/tazobactam and those receiving other treatments. Importantly, ceftolozane/tazobactam exhibited a significantly reduced risk of acute kidney injury compared with polymyxin/aminoglycoside.^129^ Also, a recent multicentre study from 2024 led by Hareza showed the emergence of ceftolozane/tazobactam resistance in patients presenting DTR *P. aeruginosa* infections.^130^ In this study, prolonged treatment and continuous renal replacement therapy were identified as relevant factors contributing to resistance emergence.^130^

### Eravacycline

Eravacycline is a synthetic fluorocycline antibiotic designed for treatment of cIAI. As a tetracycline derivative, eravacycline binds the bacterial ribosomal 30S subunit, disrupting protein synthesis. However, key structural modifications in its tetracyclic D ring increase its stability and efficacy against a wide antibacterial spectrum, including multidrug-resistant Gram positive (MDR-GP) and MDR-GN bacteria, such as MRSA, VRE, MDR *A. baumannii*, and KPC- and ESBL-producing Enterobacterales.^131^ These structural modifications allow eravacycline to bind ribosomal subunits with a 10 times higher affinity and inhibit protein synthesis to a greater extent with smaller antibiotic concentrations, compared with traditional tetracycline.^132^ In 2018, eravacycline was approved by the FDA and EMA for use in the USA and EU, respectively.^133,134^ Multiple studies have demonstrated eravacycline *in vitro* activity against Gram-positive and GN bacteria, and MDR pathogens associated with cIAIs.^132^  *In vitro* susceptibility studies have confirmed broad eravacycline activity against Gram-positive and GN pathogens, including MRSA, VRE, CRE and CRAB isolates, although *P. aeruginosa* is intrinsically non-susceptible. Three major RCTs have been performed to evaluate eravacycline efficacy *in vivo*. Phase 2 and Phase 3 (IGNITE1) trials compared eravacycline and ertapenem treatments in hospitalized patients with cIAIs, caused mainly by GN pathogens. In both studies, eravacycline showed similar clinical success compared with ertapenem, with clinical cure rates of up to 100% and 92%, respectively, in the Phase 2 trial, and 87% and 88%, respectively, in the IGNITE1 trial.^135,136^ The Phase 3, multicentre RCT (IGNITE4) compared eravacycline and meropenem in 500 hospitalized patients with cIAIs. CRE were identified only in patients treated with meropenem, while most of the identified pathogens among treatment groups contained ESBL types. Eravacycline proved to be non-inferior to meropenem as regards clinical cure rates at 25–31 trial days (90% and 91%, respectively), and clinical cure rates were similar for both treatments also in patients infected with ESBL-producing Enterobacterales (87% and 84% in eravacycline and meropenem, respectively). Low rates of adverse events were registered for eravacycline, proving it to be a safe approach for cIAIs.^137^ Interestingly, multiple real-world studies confirmed eravacycline efficacy not only for treatment of cIAIs, but also for other clinical conditions. A recent observational study investigated outcomes of over 400 patients in 19 US hospitals with respiratory infections and BSIs treated with eravacycline. Clinical success was achieved by 75% of patients, and the infections were eradicated in 94% of patients within 30-days.^138^ Similarly, a multicentre study showed that eravacycline was effective in 66 patients presenting with intrabdominal infections (IAIs), pulmonary and skin/soft tissue infections, with 95% of patients achieving clinical improvement and 86% of patients eradicating the infection.^139^ Considering the high relevance of eravacycline use against CRAB, its efficacy *in vivo* was investigated. Among 46 patients presenting with pneumonia, IAIs, skin infections or bacteraemia associated with *A. baumannii* and treated with eravacycline, the 30 day mortality rate was 23% for susceptible *A. baumannii*, and 21% for CRAB.^140^

According to these studies, eravacycline is a valuable choice for treatment of multiple difficult-to-treat and resistant pathogens causing cIAIs.^141^

### Sulbactam/durlobactam

Sulbactam/durlobactam is a combination of two β-lactamase inhibitors specifically developed for the treatment of *A. baumannii* complex (ABC) infections, including CRAB.^142^ This new treatment includes sulbactam, a classical class A β-lactamase inhibitor that targets PBPs, thus carrying crucial antibacterial properties against ABC spp.; and durlobactam, a β-lactamase inhibitor, active against a broad range of serine β-lactamases, including OXA derivatives with carbapenemase activity found in *A. baumannii*.^143,144^ Sulbactam is commonly susceptible to β-lactamases expressed by ABC; however, durlobactam prevents sulbactam degradation, restoring its activity and making the sulbactam/durlobactam combination effective against carbapenem-resistant *A. baumannii calcolaceticus* complex (CR-ABC).^145^ Following results from the Phase 3 registration trial (ATTACK), in May 2023 sulbactam/durlobactam was approved by the FDA for treatment of ABC-associated HAP/VAP in patients over 18 years old.^143^

The Phase 3 ATTACK trial compared sulbactam/durlobactam with colistin treatments, both in combination with imipenem/cilastatin, in patients with HAP/VAP associated with CRAB infections. Indeed, 96% of the 176 isolates were resistant to carbapenems. Sulbactam/durlobactam was found to be non-inferior to colistin treatment in the 28 day all-cause mortality outcome (19% and 32.3%, respectively).^146^

### Cefepime/enmetazobactam

Cefepime/enmetazobactam is a new β-lactam/β-lactamase inhibitor combination antibiotic against MDR-GN pathogens for treatment of cUTI, HAP/VAP and bacteraemia. Cefepime is a widely used β-lactam antibiotic; it binds PBPs and inhibits cell wall synthesis, and is resistant to hydrolysis by AmpC. Enmetazobactam inhibits ESBLs.^147^ In February 2024, cefepime/enmetazobactam was approved by the FDA for cUTI treatment in adults, and in March 2024 it was approved by the EMA for treatment of cUTI, HAP/VAP and bacteraemia in adults.^148^ A Phase 3, non-inferiority RCT compared cefepime/enmetazobactam with piperacillin/tazobactam treatments in more than 1000 patients worldwide presenting with cUTIs caused by GN pathogens. All patients received a 2 h infusion every 8 h. Following a 7 day course, cefepime/enmetazobactam proved to be superior for cUTI treatment, with 79% of patients treated with cefepime/enmetazobactam achieving clinical cure and infection eradication (<10^3^ cfu/mL in urine), compared with 58% of patients treated with piperacillin/tazobactam. Both treatments showed a similar safety profile, with mild adverse events reported by 50% and 44% of patients treated with cefepime/enmetazobactam or piperacillin/tazobactam, respectively.^149^

### Aztreonam/avibactam

Aztreonam/avibactam is a novel β-lactam/β-lactamase inhibitor combination antibiotic, which marries avibactam activity to the effects of the traditional antibiotic aztreonam. Aztreonam binds PBPs, inhibiting cell wall synthesis in GN bacteria, but it can be hydrolysed by MBL-producing pathogens. Avibactam inhibits several Ambler class A, C and D enzymes, such as ESBLs, AmpC enzymes and KPC, thus restoring aztreonam activity against MBL-GN.^150^ In April 2024, aztreonam/avibactam was approved by the EMA for treatment of HAP/VAP and difficult-to-treat cIAI caused by Enterobacterales and *P. aeruginosa*.^151^ A systematic review from 2021 showed that aztreonam/avibactam presented a high antimicrobial activity (MIC ≤4 mg/L) in 52.8% of isolates, most of which were MBL-producing Enterobacterales (79%) and *Stenotrophomonas maltophilia* (85%). However, low antimicrobial activity (MIC >4 mg/L) was registered for *P. aeruginosa* isolates, which may require different antibiotic approaches.^150^ A Phase 3 RCT (REVISIT) compared aztreonam/avibactam with meropenem treatments in 422 hospitalized patients with HAP/VAP and serious IAIs caused by GN bacteria. Aztreonam/avibactam proved to be an effective therapy, with 68% of treated patients achieving clinical cure at 25–31 trial days, compared with 66% of patients treated with meropenem. Additionally, aztreonam/avibactam resulted in lower 28 day mortality rates compared with meropenem (4% and 7%, respectively) and proved to be safe, with no serious treatment-related adverse events.^152^

Aztreonam/avibactam (Emblaveo) was subsequently approved by the FDA in February 2025 for the treatment of IAI and HAP/VAP in patients with limited or no alternative treatment options. Reflecting this and accumulating comparative data, the 2026 IDSA guidance on the treatment of antimicrobial-resistant GN infections now designates aztreonam/avibactam, alongside cefiderocol, as a preferred agent for infections caused by NDM-producing Enterobacterales, with the combination of ceftazidime/avibactam plus aztreonam reserved as an alternative when aztreonam/avibactam is unavailable.^43^

### Cefepime/taniborbactam

Cefepime/taniborbactam is an investigational β-lactam/β-lactamase inhibitor combination antibiotic developed for treatment of cUTIs and HAP/VAP caused by CRE and MDR *P. aeruginosa*.^153^

Cefepime is effective against susceptible GN bacteria, by binding PBPs, while taniborbactam is an inhibitor of serine β-lactamases and MBLs. *In vitro* studies proved that cefepime is more effective when used in combination with taniborbactam, as the latter is able to restore cefepime’s activities against MDR pathogens, such as CRE and CRPA.^154,155^ A worldwide surveillance study from 2018 to 2022 (GEARS) tested the *in vitro* activity of cefepime/taniborbactam compared with cefepime treatment alone in Enterobacterales and *P. aeruginosa* isolates. Compared with cefepime alone, cefepime/taniborbactam registered a >64-fold reduction of MIC_90_ values against Enterobacterales (>16–0.25 mg/L) and a 4-fold reduction against *P. aeruginosa* isolates (32–8 mg/L), showing a great inhibition effect on CR isolates.^154^

A Phase 3 RCT compared IV treatment with infusions of cefepime/taniborbactam or meropenem in hospitalized patients with cUTIs caused by Enterobacterales and *P. aeruginosa.* Both treatments showed similar safety profiles, with adverse events registered in 35.5% and 29.0% of patients treated with cefepime/taniborbactam and meropenem, respectively.^156^ According to this trial, cefepime/taniborbactam treatment proved to be superior to meropenem in primary outcomes measuring microbiological and clinical success at 19–23-days of trial.

### Caveat for sepsis/septic shock patients

Notably, there are limited data about the clinical efficacy of each above-mentioned new antibiotic in patients treated for sepsis or septic shock in the ICU. For instance, data from pivotal trials in patients with cUTI cannot be transferred reliably to CIPs. Even for non-inferiority trials in HAP, many participants did not suffer from sepsis and/or septic shock. This limitation should be taken into account for a careful interpretation of the above-mentioned clinical trials conducted with new antibiotics.

## Dosing strategies and barriers to the implementation of new antibiotics

Currently, optimal dosing strategies for some of these molecules, particularly for fragile patients or CIPs, including those in ICUs, are not yet well established. The development of rapid bedside diagnostic tests, capable of identifying pathogens and detecting their resistance mechanisms, is anticipated in the near future. These advancements, along with the accumulation of real-world clinical data, are expected to shed light on the efficacy of these treatments and the risk of developing resistance, thereby guiding more effective and evidence-based therapeutic decisions.^7^

In a study by Bavaro *et al.*^157^ from 2023, the implementation of the loading dose strategy in combination with continuous infusion of new β-lactam/β-lactamase inhibitor combinations in hospitalized patients with GNB BSIs was associated with better outcomes and a lower mortality risk in immunocompromised patients and in patients in septic shock, compared with patients treated with the intermittent bolus strategy. In general, the aforementioned combination strategies of β-lactam/β-lactamase inhibitors or combination of different antibiotics are regarded as an optimal strategy for treatment of MDR-GNB infections, although current evidence is still limited to studies with small samples.^158^ A survey from 2022 investigated the preferred treatment strategies of over 1000 clinicians worldwide for CR-GNB infections and highlighted that a standardized strategy for combination treatments of MDR-GNB is still far from being achieved.^159^ In the presence of a severe clinical condition, an empirical coverage for CR-GNB was preferred by 80% of clinicians from high-income countries, while only half of clinicians from lower-income countries adopted this strategy, mostly due to the limited therapeutic options available. Combination treatments were the preferred strategy in targeted treatments; however, a great variability of combinations was identified, with 40 different regimens adopted for CRAB and over 100 regimens in CRE, with carbapenem/polymyxin being the most prescribed in patients with sepsis, independently from the pathogen.^159^

The lack of a standardized combination strategy was also evident in this survey, as clinicians reported different definitions of combination therapy, such as simple combinations of compounds, combination of compounds with *in vitro* activity, and combination of compounds with *in vitro* synergism.^159^ Discrepancies in combination therapy definitions have been identified in several clinical studies, making the results difficult to generalize.^160^

Despite the potential of these new antibiotics, several barriers hinder their widespread adoption. These include selective testing and reporting in antibiograms, limited availability and accessibility, high costs, and concerns over fostering resistance. The lack of rapid diagnostic tools at the point of care exacerbates these challenges, making it difficult for clinicians to make informed decisions about empirical therapy (Table 2).

**Table 2.** Barriers to accessing and prescribing new antibiotics

Issue	Description
Lack of early pathogen identification tools	Rapid diagnostic testing at the POC is often lacking, making it difficult to promptly identify the causative pathogen, which is crucial for empirically using new antibiotics.^6,161^
Selective testing and antibiograms	Comprehensive testing of new antibiotics against various pathogens may be limited, leading to knowledge gaps regarding their effectiveness. Effective AST can be challenging for recently approved antibiotics due to delayed availability of clinical breakpoints and inclusion in commercial panels or access to other *in vitro* susceptibility materials.^162,163^
Limited availability and antibiotic shortages	Newly developed antibiotics may not be readily available in all regions or hospitals, especially if they are not included in local formularies or pharmacies do not stock them.^164,165^ Frequent antibiotic shortages in European hospitals have affected over 80% of hospital pharmacists in some years, primarily managed through substitution. Shortages can challenge antimicrobial stewardship efforts and contribute to suboptimal patient outcomes and resistance.^165,166^
Restricted accessibility	Institutional protocols may require approval from infectious disease specialists before prescribing new antibiotics in emergency departments and ICUs, which can be problematic in urgent situations like sepsis.^167–169^
Pricing and cost-centric decision making	High costs associated with new antibiotics and inadequate reimbursement policies can limit their clinical use.^161,170,171^
Fear of generating resistance	Concerns about rapid resistance development to new antibiotics may lead to hesitancy in their use, despite the potential risks of not prescribing antibiotics when needed.^172,173^

AST, antimicrobial susceptibility testing; POC, point of care.

According to clinicians worldwide, standard susceptibility tests are generally available at local levels, independently of income status. In contrast, in resource-limited settings (RLSs) complex diagnostic tests, such as MALDI-TOF and nucleic acid amplification tests (NAATs), are not easily accessible. Using external laboratories is usually an alternative solution. Lack of testing availability in RLSs results in extended duration of diagnosis compared with high-income countries, with only 23% of clinicians in RLSs receiving blood cultures more than 72 h of sampling, against 7% in high-income countries.^159^ Considering that in many Asian and African RLSs it is common to acquire MDR-GNB, especially for children, and patients infected with susceptible or resistant pathogens present similar clinical manifestations, it is essential to integrate laboratories and testing resources in RLSs, in order to identify the presence of resistant genes and employ the appropriate treatment and de-escalation strategies.^174^

Also, the focus on drug acquisition costs often overshadows the broader considerations of effectiveness and overall patient management costs (Table 2). It was indeed estimated that implementing newly developed antibiotics for MDR infections results in significant large incremental costs.^175^ For comparison purposes, ceftazidime/avibactam registered the highest sales 2 years post-launch (approximately $80 million), while non-antibiotic drugs registered sales in a range of $500 million to $1 billion.^176^ The cost-related limitations of implementing new antibiotics in routine treatment strategies arealso due to the high economic discrepancies between new and old antibiotics. The CDC showed that in US hospitals a 14 day treatment course for CRE or CRPA with any of the new antibiotics would incur double the cost of the same treatment with traditional antibiotics, such as colistin/tigecycline combination. The estimated costs of a treatment course for CRAB with new antibiotics would be 20 times higher, compared with the traditional colistin monotherapy.^175^ The estimated annual costs of treating MDR-GNB with new antibiotics is in a range from $30 million to over $500 million.^175^

Limited profitability is one of the main factors influencing antibiotics availability, especially in RLSs.^164^ International surveys showed that new β-lactam/β-lactamase inhibitors were available in only half of countries worldwide. Interestingly, limited availability was registered in these countries also for some ‘old’ antibiotics, such as colistin and polymyxin B.^159^ Another critical factor is the lack of updated guidelines on the use of new antibiotics in several countries, as many clinicians tend strictly to follow official recommendations, without a critical analysis of MDR risks in their patient population.^164^

Shortages of antibiotics experienced in several countries and healthcare facilities may be highly detrimental and cause a setback in the management of MDR infections. It is important to be aware that antibiotic shortages do not affect only low-income countries.^177^ A cefepime shortage in Swiss hospitals between 2005 and 2008 led to an increased use of broad-spectrum antibiotics, which resulted in a significant negative impact on *P. aeruginosa* susceptibility.^178^ One of the proposed strategies to overcome these limitations is to extend the use of old antibiotics for at least 1 year after the expiry dates in the clinical practice.^177^

With the objective of achieving responsible use of antibiotics, institutional restrictions have been implemented. Restricted antibiotics may therefore need the approval of specialists to be used in clinical practice. However, restrictive antibiotic availability may cause serious delay in treating CIPs and it may facilitate the emergence of MDR pathogens, following alternative non-appropriate treatments.^167,179^ It is therefore crucial to find a balance between excessive and irrational antibiotic use and restrictive policies, which may deny accessibility, especially in low-income countries.^180^

The limited access to or availability of effective antibiotics poses a dual risk: it jeopardizes patient care and contributes to the development of antibiotic resistance (Table 2).

## Competing AMS strategies regarding prescription of new antibiotics

AMS strategies aim to combat the escalating challenge of MDR infections, enhance patient outcomes, and decrease healthcare costs. At the heart of AMS lies the principle of using antibiotics wisely and appropriately.^181^ This goal encompasses minimizing the unintended consequences associated with antibiotic use. These include reducing the risk of toxicity, preventing the selection of pathogenic bacteria like *C. difficile*, and curbing the emergence of AMR.^182–185^

The evaluation of AMS strategies encompasses both process and outcome measures.^181,186^ Process measures evaluate the quality of care and include parameters such as adherence to prescribing guidelines, appropriate antibiotic selection, timely de-escalation, defined daily doses (DDDs) and the number of antibiotic days.^181,186–188^

Outcome measures evaluate the overall impact of AMS on patient health and healthcare system efficiency and include metrics such as reductions in AMR rates, decreased HAIs, improved clinical outcomes, lowered mortality rates, minimization of adverse effects and reduced LOS.^181,184,187^ Antimicrobial-free days is another important measure, focusing on whether patients are receiving antimicrobials or not. This metric offers a broader perspective on the impact of AMS programmes.^186,187^

Finally, regarding cost metrics, a 2019 systematic review by Nathwani and colleagues^181^ aimed at evaluating the economic and clinical impacts of AMS strategies from 146 studies, found a decrease or no change in LOS (85%) and antibiotic expenditure (92%). The mean cost savings varied by hospital size and region, with US studies showing an average saving of $732 per patient (ranging from $2.50 to $2640). European studies exhibited similar trends.

In general, AMS programmes within ICUs should be implemented in a precise and coordinated manner, to guarantee safety of CIPs and improve outcomes. Ture *et al*.^189^ in 2023 summarized the recommended AMS practices to follow within ICUs before, during and after treatment.

During treatment, PK/PD properties of antimicrobial treatment should be carefully evaluated to determine the appropriate dosage in ICU patients.^190^ Infection source control should be implemented, as it was demonstrated that it can improve outcomes to a greater extent than early antimicrobial treatment.^191^ Patients should be evaluated daily through clinical and laboratory examinations to detect changes relevant to their therapy plan, and de-escalation of broad-spectrum antibiotics should be employed immediately upon pathogen identification.^190,192^ A special population is represented by elderly patients where PK/PD of antibiotics is less predictable and risk of toxicity increased; in this setting a multifaceted organized intervention, involving both clinicians and nursing staff, should be advocated to improve antimicrobial prescriptions and minimize detrimental effects of therapy.^193,194^

## Restriction versus enablement strategy in CIPs suffering from MDR-GN infections

In AMS, two primary strategies are utilized: ‘restriction’ and ‘enablement’ (Table 3). The restriction strategy (pre-prescription approval) focuses on limiting antibiotic use to essential cases, thereby mitigating the risk of resistance. It involves stringent regulations and oversight.^195^ For example, this strategy may include measures such as requiring pre-approval from an infectious diseases (ID) specialist before certain antibiotics can be prescribed, or implementing hospital-wide policies that restrict the use of specific antibiotics to prevent overuse.^195,196^ However, in specific clinical scenarios, such as patients with septic shock or those at risk for infections by MDR-GN, the immediate use of restricted antibiotics is recommended, pending the results of culture tests, to ensure prompt and effective treatment.^13,196^

**Table 3.** Pros and cons for the restriction versus enablement strategies

Aspect	Restriction strategy	Enablement strategy
Focus	Limiting antibiotic use to essential cases	Ensuring beneficial antibiotics are accessible, promoting informed decision-making
Approach	Pre-approval from ID specialist, hospital-wide policies restricting specific antibiotics	Real-time consultations with ID experts, decision-support tools
PROS	Reduces broad-spectrum antibiotic use and antimicrobial resistance Optimizes initial antibiotic choices Controls antibiotic use through prescribing barriers Reduces costs Reduced duration of antibiotic administration	Improves clinical support for prescribers Offers flexibility in timing Provides educational opportunities Maintains prescribers’ decision-making autonomy Reduces costs Reduced duration of antibiotic administration Adaptability to individual patient needs
CONS	Limits prescribers’ autonomy and negatively impacts the professional culture May increase use of non-restricted antibiotics Time-consuming pre-authorization process Requires highly skilled and available ID physician for approvals May delay necessary treatments	Generally labour intensive Requires highly skilled physician for education

ID, infectious diseases.

Observational studies have shown the efficacy of prescription restrictions in reducing broad-spectrum antibiotic use and AMR.^195,197^ This approach is particularly relevant for new antibiotics or specific patient groups.^45,198–201^ For example, implementing a restrictive antibiotic approach during allogeneic stem cell transplantation (SCT) has shown significant benefits in patient care and microbiome preservation in a single-centre study (University Hospital in Regensburg). The structure transitioned from a permissive antibiotic strategy, which involved initiating antibiotics for all neutropenic fever patients, to a more selective approach that focused on cases with a high likelihood of cytokine release syndrome, particularly after anti-thymocyte globulin therapy. The restrictive antibiotic approach postponed the start of antibiotic administration from 1.4 ± 7.6 days before SCT to 1.7 ± 5.5 days after SCT and significantly reduced the duration of antibiotic administration by 5.8 days (*P* < 0.001) without an increase in infectious complications. It also led to improvements in microbiome diversity, with a positive trend towards a reduced incidence of severe gastrointestinal graft-versus-host disease.^201^

Advantages of this approach may include minimizing the misuse or overuse of antibiotics, optimizing initial antibiotic choices, indirectly controlling antibiotic use through prescribing barriers, reducing costs, and offering a chance to educate prescribers about correct antimicrobial therapy practices.^184,195,196^

This approach, while effective in many ways, does have its drawbacks. One major concern is the potential limitation it places on the autonomy of prescribers in making clinical decisions.^167,195,196^ Additionally, this method might unintentionally cause an increase in the use of non-restricted antibiotics. Furthermore, the pre-authorization process can be quite time-consuming and necessitates a subsequent review of the patient's chart.^196^ It is also crucial that the ID physician in charge of approvals is not only highly skilled and qualified but also readily available to make timely decisions.^196,202^ The enablement strategy (education, post-prescription feedback and audit), conversely, aims to ensure that beneficial antibiotics are accessible when needed, promoting informed decision-making among healthcare professionals. For example, this could involve real-time consultations with ID experts or the use of decision-support tools that help clinicians select the most appropriate antibiotic based on a patient's specific situation.^195,203^ Utilizing this approach provides several unique benefits. It offers improved clinical support for those who prescribe medications, allows for greater flexibility in terms of timing, and provides educational opportunities. Importantly, it achieves all this without compromising the prescribers’ independence in making decisions.^195,204,205^ A drawback of interventions that focus on enablement is that they typically require a significant amount of workforce and expertise.^206^

The Cochrane systematic review and meta-analysis by Davey *et al.*^195^ on AMS strategies highlighted the effectiveness of both these strategies in improving antibiotic prescribing for hospitalized patients. The study included both randomized (*n* = 58) and non-randomized trials (*n *= 163), with 183 from North America and Europe; it found that these interventions reduced the duration of antibiotic treatment by an average of 1.95 days from 11 days, based on high-certainty evidence from 14 RCTs. Non-randomized studies echoed these findings, showing improvements in prescribing practices in routine clinical settings. Notably, most of these interventions were implemented hospital wide. In terms of patient outcomes, the risk of death was similar in both intervention and control groups (11% each), suggesting that reducing antibiotic use does not adversely affect mortality rates. Additionally, AMS interventions are likely to reduce the LOS by about 1.12 days, based on moderate-certainty evidence from 15 RCTs.^195^ Thus, the Cochrane review, while acknowledging the effectiveness of restrictive interventions in AMS programmes, also revealed some critical concerns. It pointed out the possibility that restrictive interventions could delay necessary treatments and negatively impact the professional culture within healthcare settings due to diminished communication and trust between ID specialists and clinical teams. This aspect of the review emphasized the complex balance that must be maintained in AMS. Furthermore, the review emphasized the necessity for further research to explore and understand the unintended consequences of restrictive interventions.^195^

In parallel, a study conducted by Sulis *et al.*^207^ in 2022 examined the relationship between antibiotic exposure and the emergence of multi-drug resistant organisms (MDROs). This research, encompassing all three AWaRe (Access, Watch, Reserve) categories from the WHO’s strategy—which categorizes antibiotics to inform their appropriate use and mitigate resistance—revealed significant associations between antibiotic usage and MDRO development. Notably, the findings showed that Watch and Reserve antibiotics were associated with higher ORs for MDRO colonization or infection. This outcome raises questions about the efficacy of overly stringent restrictions on Reserve antibiotics, suggesting that the development of resistance is a widespread issue, impacting all antibiotic categories, including Access and Watch. Thus, these studies collectively highlight the nuanced challenges in formulating and implementing antibiotic policies, highlighting the need for a balanced, evidence-based approach in antibiotic stewardship.

## Authors’ agreement for enablement strategy of new antibiotics in severe MDR-GN infections

The following section reflects the collective clinical opinion and consensus of the authors rather than a direct synthesis of primary evidence and should be interpreted accordingly. The authors of this review support the use of new antibiotics in CIPs suffering from MDR-GN infections as recommended in the recent international guidelines from the IDSA and ESCMID. However, already available data from the USA have shown that despite these guidelines, clinicians under-prescribe new anti-infectives for MDR pathogens.^208^ This might compromise the care of the patients and is against the stewardship rules of hitting hard, hitting fast when faced with a CIP.

To implement the optimal treatment strategy, we propose here an algorithm to support and coordinate AMS programmes and teams within ICUs in the case of sepsis or septic shock in CIPs. The authors recommend the implementation of this tool in the routine standard operating procedure, to optimize outcomes for CIPs (Figure 2).

Therefore, the authors encourage an enablement strategy for CIPs suffering from MDR-GN infections to reduce mortality and to reduce toxicity of using the ‘old’ antibiotics. The authors recommend that this strategy, which is meant as an exception applicable only for CIPs, be embedded in local AMS programmes under the education and lead of experienced clinicians.

## Conclusions

AMR poses a substantial challenge to public health, economies and healthcare systems worldwide. Clinically, AMR leads to an increased prevalence of infections that are DTR, contributing to higher mortality rates. Particularly, infections caused by MDR-GN are correlated with higher mortality rates compared with infections caused by susceptible strains in CIPs and in ICUs. ‘Old’ antibiotics are correlated with reduced cure rates and increased toxicity in patients with infections due to CROs. International guidelines recommend the initial use of new antibiotics for CRO infections. An enablement strategy for CIPs suffering from MDR-GN infections facilitating the prescription of new antibiotics can reduce mortality and toxicity. This strategy should be applicable only for CIPs and should be embedded in local AMS programmes.

## Transparency declarations

R.C. has participated in educational programmes sponsored by Advanz Pharma, GSK, Menarini, MSD, Pfizer and Shionogi, and has received research grants funded by Pfizer and Shionogi. M.Antonellihas received a research grant from GE, and board participation from Shionogi, Menarini and Novartis in the last 3 years. P.M. has received fees for lectures and participation on advisory boards for Menarini, MSD, Pfizer, Viatris and Shionogi. M. Akova has received research grants from Pfizer and Gilead, in both cases paid to his institution. A.S. has received fees for advisory boards or lectures from Pfizer, Menarini, Shionogi, Advance Pharma, and grants from Pfizer, AdvanzPharma and Gilead. S.S.K. has received speaker and advisory board honoraria from Pfizer, MSD, Menarini, Gilead, Hikma and Antabio, for antibacterial agents. J-F.T. reports lectures for bioMérieux, Merck, Pfizer, Shionogi, Advanz, Tillotts and Gilead, advisory board participation for Menarini, Gilead and Roche Diagnostic, and a research grant to his research unit from Merck. M.F. has participated in lectures and/or the advisory board for Pfizer, Menarini, GSK, Advanz Pharma, Meji Pharma, Tillotts and Shionogi. M.G. discloses participation in advisory boards for Pfizer, Menarini, Advanz and Napp. All other authors report no conflicts of interest.
